# Familial Cerebral Cavernous Malformations: Pathophysiology, Genetics, Biomarkers, and Treatment Perspectives

**DOI:** 10.1111/jnc.70342

**Published:** 2026-01-04

**Authors:** Fabrícia Lima Fontes‐Dantas, Gustavo da Fontoura Galvão, Alexandre Martins Cunha, Pedro de Sena Murteira Pinheiro, Verônica Morandi, Jorge Marcondes de Souza

**Affiliations:** ^1^ Neuropharmacogenetics Laboratory, Department of Pharmacology and Psychobiology, Roberto Alcantara Gomes Institute Biology (IBRAG) Rio de Janeiro State University (UERJ) Rio de Janeiro Brazil; ^2^ Department of Neurosurgery Clementino Fraga Filho University Hospital, Federal University of Rio de Janeiro (UFRJ) Rio de Janeiro Brazil; ^3^ Department of Surgical Specialties, Neurosurgery Division Pedro Ernesto University Hospital, Rio de Janeiro State University (UERJ) Rio de Janeiro Brazil; ^4^ Laboratory of Evaluation and Synthesis of Bioactive Substances (LASSBio), Institute of Biomedical Sciences Federal University of Rio de Janeiro (UFRJ) Rio de Janeiro Brazil; ^5^ Laboratory of Endothelial Cell Biology and Angiogenesis (LabAngio), Department of Cell Biology, Roberto Alcantara Gomes Institute Biology (IBRAG) Rio de Janeiro State University (UERJ) Rio de Janeiro Brazil

**Keywords:** cerebral cavernous malformation, FCCM, genetic, genetic counseling, magnetic resonance sequence, quality of life, RhoA/ROCK signaling

## Abstract

Familial cerebral cavernous malformations (FCCM) are a heritable neurovascular disorder defined by clusters of dilated, thin‐walled capillaries in the brain and spinal cord. Although rare, FCCM offers a tractable model for understanding how genetic disruptions in endothelial junction biology, mechanotransduction, and kinase signaling drive vascular instability in the central nervous system. Pathogenic loss‐of‐function variants converge on signaling abnormalities that promote barrier dysfunction, iron deposition, inflammation, and progressive lesional growth. Clinically, FCCM may manifest with seizures, headaches, focal deficits, or intracerebral hemorrhage, yet many carriers remain asymptomatic owing to incomplete and age‐dependent penetrance. Advances in neuroimaging have enhanced the detection of micro‐lesions and iron accumulation, establishing these modalities as central biomarkers of disease expression. Complementing imaging, emerging circulating biomarkers, including inflammatory cytokines and plasma microRNAs associated with mutation status, may improve individualized risk stratification. This primer synthesizes current knowledge on FCCM pathophysiology, genetics, diagnostic strategies, and therapeutic perspectives. By integrating molecular mechanisms with clinical relevance, it outlines a framework for understanding FCCM as a disorder of perturbed endothelial signaling and neurovascular homeostasis, and highlights opportunities to advance precision medicine for this challenging condition.

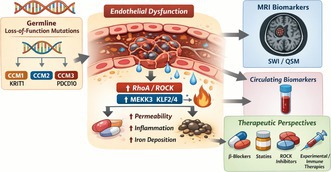

## Graphical abstract

Cerebral cavernous malformations (CCMs) are vascular lesions in the brain caused by inherited genetic mutations in the CCM1/2/3 genes that disrupt normal blood vessel function. This work demonstrates that these mutations lead to endothelial dysfunction, inflammation, and iron accumulation, which can be detected by magnetic resonance imaging (MRI) and by circulating biomarkers. The study also highlights current and emerging therapeutic strategies that target these molecular pathways to improve diagnosis and treatment. SWI/QSM: susceptibility‐weighted imaging/quantitative susceptibility mapping.

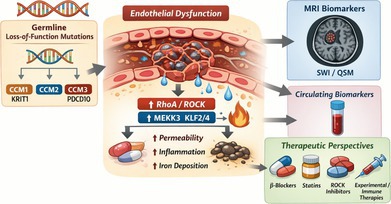

**Abbreviations**
AKT/Akt: protein kinase B
ANG2: angiopoietin 2
BNDF: brain‐derived neurotrophic factor
CCL: C–C motif chemokine ligand
CCM: cerebral cavernous malformation
CD: cluster of differentiation (several numbered isoforms)
CDCP1: CUB domain–containing protein 1
Cdh5: cadherin 5
CNV: copy number variation
CreERT2: tamoxifen‐inducible Cre recombinase
CRISPR: clustered regularly interspaced short palindromic repeats
CRP: C‐reactive protein
CRT: calreticulin
CXCL: C–X–C motif chemokine ligand
DCE: difficult‐to‐control epilepsy
DVA: developmental venous anomaly
ECM: extracellular matrix
EndoMT: endothelial‐to‐mesenchymal transition
EQ‐5D‐5L: five‐level EuroQol five‐dimension questionnaire
ERK5: extracellular signal‐regulated kinase 5
FCCM: familial cerebral cavernous malformations
FGF‐21: fibroblast growth factor 21
FLT3L: Fms‐like tyrosine kinase 3 ligand
FN: fibronectin
GCKIII: germinal center kinase III
GDF‐15: growth differentiation factor 15
GOF: gain of function
hBBB: human blood–brain barrier
HRQoL: health‐related quality of life
ICAM‐1: intercellular adhesion molecule 1
ICAP‐1: integrin cytoplasmic domain–associated protein 1
IL: interleukin
iPSC/hiPSC: induced pluripotent stem cells/human induced pluripotent stem cells
IVF: in vitro fertilization
JAK: janus kinase
KLF2/4: Krüppel‐like factor 2/4
KRIT1: krev interaction trapped 1
LBP: lipopolysaccharide‐binding protein
LOF: loss of function
MEK5: mitogen‐activated protein kinase kinase 5
MEKK3: mitogen‐activated protein kinase kinase kinase 3
MGC4607: CCM2 (malcavernin)
MLPA: multiplex ligation‐dependent probe amplification
MMP: matrix metalloproteinases (several numbered isoforms)
MRI: magnetic resonance imaging
Msh2: MutS homolog 2
MST3: mammalian Ste20‐like kinase 3
MST4: mammalian Ste20‐like kinase 4
NGS: next‐generation sequencing
NPxY: Asparagine–proline–X–tyrosine motif
NSAIDs: non‐steroidal anti‐inflammatory drugs
OMIM: online mendelian inheritance in man
PAI‐1: plasminogen activator inhibitor 1
PDCD10: programmed cell death 10
PEG3: paternally expressed gene 3
PIK3CA: phosphatidylinositol‐4,5‐bisphosphate 3‐kinase catalytic subunit alpha
PROMIS‐29: patient‐reported outcomes measurement information system 29
PROs: patient‐reported outcomes
QSM: quantitative susceptibility mapping
Rac1: Ras‐related C3 botulinum toxin substrate 1
Rap1: Ras‐related protein 1
RhoA/ROCK: ras homolog family member A/Rho‐associated protein kinase
RICM: reflection interference contrast microscopy
RTK: receptor tyrosine kinase
SCCM: sporadic cerebral cavernous malformation
sENG: soluble endoglin
Slco1c1: solute carrier organic anion transporter family member 1C1
sROBO4: soluble roundabout guidance receptor 4
STK25: serine/threonine kinase 25
STRIPAK: striatin‐interacting phosphatase and kinase complex
SWAN: susceptibility‐weighted angiography
SWI: susceptibility‐weighted imaging
T2* GRE: T2*‐weighted gradient echo
Th17/Tc17: T helper 17/cytotoxic T 17 cells
THBS1: thrombospondin 1 gene
TLR: toll‐like receptor
Trp53: Tumor protein p53
TSP‐1: Thrombospondin 1 protein
VE‐cadherin: vascular endothelial cadherin
VEGF: vascular endothelial growth factor
VEGFR2: vascular endothelial growth factor receptor 2

## 1 Introduction

Cerebral cavernous malformations (CCMs) are vascular malformations found in the central nervous system (CNS). These lesions consist of enlarged capillary cavities lined by a single layer of endothelium, without interposing brain parenchyma, and with loss of normal capillary angioarchitecture and hemodynamics (Awad and Polster 2019). Studies of human CCM lesions and animal models have documented increased vascular permeability and loss of endothelial junction integrity, features consistent with blood–brain barrier dysfunction (Tu et al. 2005; Stockton et al. 2010). Histopathological studies of human CCM lesions show glial and perivascular alterations, indicating that CCM pathology extends beyond endothelial cells (Clatterbuck et al. 2001). Increasing evidence suggests that impaired endothelial mechanotransduction in CCM can disrupt neurovascular‐unit function and contribute to seizure susceptibility, as in other conditions in which barrier dysfunction promotes neuronal hyperexcitability (Lisowska et al. 2018; Kutikhin et al. 2022).

The disease exhibits unique genetic features that provide insights into nervous system's vascular development, unlike those of any other cerebrovascular disease. Natural history studies suggest that sporadic CCMs are among the most prevalent CNS vascular malformations (5%–15%), affecting approximately 0.2%–0.5% of the world's population (Gross et al. 2011). However, the familial or hereditary form (FCCM), caused by autosomal‐dominant loss‐of‐function (LOF) mutations in *KRIT1/CCM1* (53%–65% in FCCM), *CCM2/MGC4607* (10%–16%), or *PDCD10/CCM3* (5%–15%) (Labauge et al. 2007; Flemming et al. 2023), constitutes a rare genetic disorder with an estimated prevalence of 1–5:10000 (Orphanet–OMIM 11860/603284/603285/619538) (Labauge et al. 2007). The three proteins encoded by CCM genes interact to form a ternary complex that stabilizes interendothelial junctions and cell‐matrix adhesion (Flemming et al. 2023).

Unlike sporadic CCM, FCCM typically presents with multiple lesions throughout the brain and spinal cord (Awad and Polster 2019). Given the exceptional genetic heterogeneity of CCM, careful imaging evaluation is essential when assessing individuals at risk for FCCM. Magnetic Resonance Imaging (MRI) remains the diagnostic gold standard, and susceptibility‐based sequences, particularly susceptibility‐weighted imaging (SWI), are strongly recommended because of their superior sensitivity in detecting numerous small lesions and punctate microhemorrhages characteristic of the familial form (de Souza et al. 2008; de Champfleur et al. 2011). Thus, the emphasis on SWI in FCCM diagnosis reflects the typical multiplicity (typical number 6–20 CCMs) and small size of lesions rather than any inherent imaging difference between familial and sporadic cavernomas (Flemming et al. 2023).

In this review, we will provide background information on the disease and its pathophysiology, including the impact of investigation and treatment on families, and highlight the role of genetic predisposition and inheritance patterns.

## 2 Clinical Features and Initial Investigation

**FIGURE 1:**
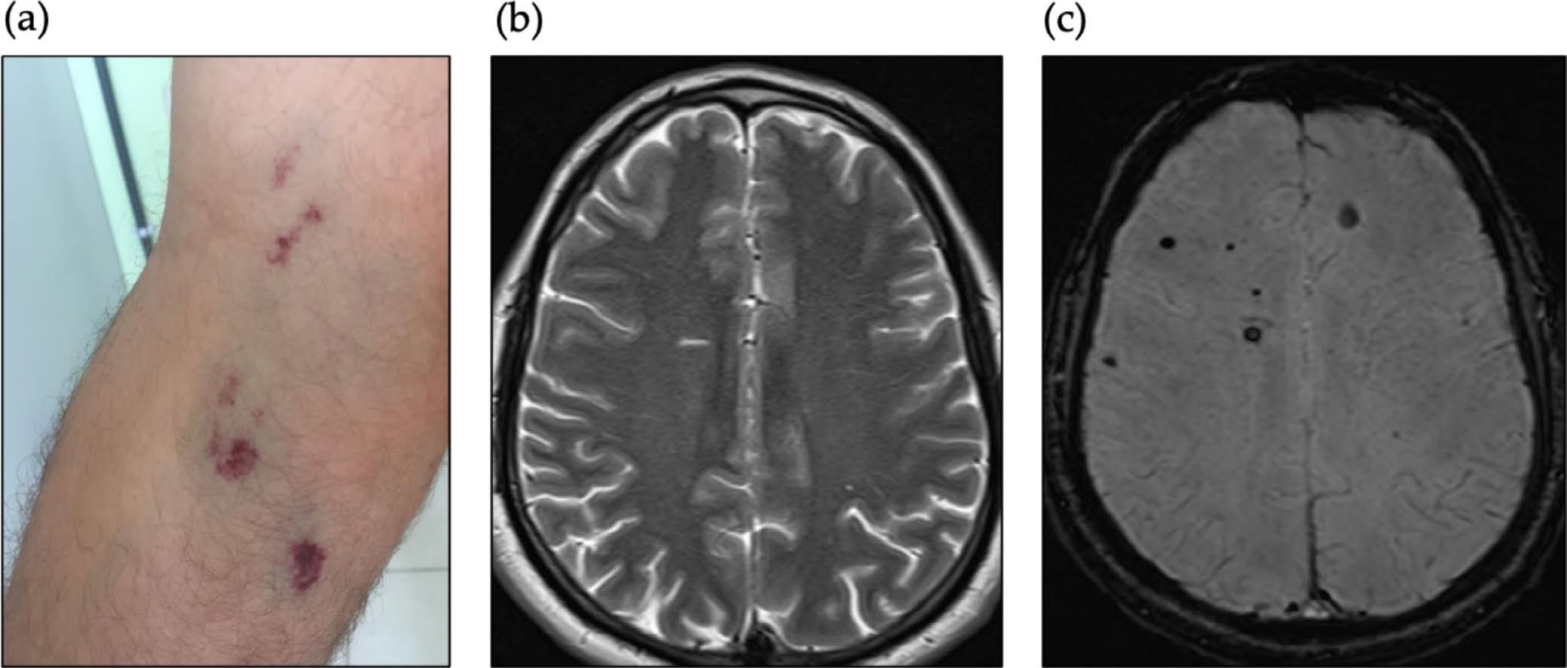
Investigation of familial cerebral cavernous malformation (FCCM) and its clinical features: (a) A 42‐year‐old asymptomatic man, whose mother is known to have multiple brain lesions, presented with multiple violaceous cutaneous lesions on the right forearm. (b) T2‐weighted axial sequence magnetic resonance imaging (MRI) of a very small lesion on the deep left parietal region of a 42‐year‐old asymptomatic man with a mother known to have multiple brain lesions. (c) A susceptibility‐weighted imaging (SWI) sequence of MRI in the same axial cut as (b), showing multiple lesions.

Patients with FCCM generally present with the same neurological manifestations seen in sporadic cases, including seizures, headaches, transient or progressive focal deficits, and symptomatic intracerebral hemorrhage (Awad and Polster 2019; Gross et al. 2011). Cutaneous vascular anomalies, such as hyperkeratotic capillary‐venous malformations, punctate capillary lesions, or dark blue nodules (Figure 1a), can provide an essential clue to FCCM, particularly when multiple cerebral lesions are present on MRI (Labauge et al. 2007; Manole et al. 2020). Additional findings reported in some families include hepatic or renal hemangiomas, intraosseous venous malformations, retinal cavernomas, and, in CCM3 cases, multiple meningiomas (Shenkar et al. 2015). Many individuals remain asymptomatic, and the diagnosis is frequently made incidentally on MRI (Figure 1b,c) performed for unrelated reasons (Akers et al. 2025). Symptoms usually arise between the 2nd and 5th decades, although onset at any age is possible. *CCM3/PDCD10* mutations are associated with earlier and more aggressive presentations, often beginning in childhood (Shenkar et al. 2015; Galvão, da Silva, et al. 2024).

Radiological imaging is a central component of the clinical investigation of suspected FCCM, however, the well‐recognized phenomenon of incomplete penetrance complicates interpretation. Two related concepts are essential for clinicians: genetic penetrance, the proportion of mutation carriers who manifest disease features, and radiological penetrance, the likelihood that a carrier will display CCM lesions detectable on MRI, independent of symptoms (Akers et al. 2025). These forms of penetrance do not always coincide, and some individuals with confirmed pathogenic variants may show no radiographic abnormalities at a given time.

It has been observed that radiological penetrance is especially age‐dependent. In *KRIT1*‐positive families, carriers may have normal MRI studies early in life and only develop lesions on later imaging, illustrating the dynamic nature of lesion appearance (Battistini et al. 2007). Although *CCM2* and *KRIT1* mutations have been classically associated with higher radiological penetrance than *PDCD10*, penetrance is not absolute and varies across genes and families (de Vos et al. 2017). Notably, Scimone et al. (2018) identified several young *CCM2* mutation carriers with completely regular MRI scans, demonstrating that radiological expression may remain absent throughout childhood and early adolescence (Scimone et al. 2018).

## 3 Pathophysiology

### 3.1 Initiation and Expansion of CCM Lesions

Compelling genetic evidence indicates that FCCM follows a “two‐hit” mechanism of lesion formation. In this model, patients inherit a germline LOF mutation in one allele of a CCM gene. Still, cavernomas arise only after a somatic second hit inactivates the remaining wild‐type allele in a subset of endothelial cells (Akers et al. 2009; Pagenstecher et al. 2009; McDonald et al. 2011; Spiegler et al. 2019; Rath et al. 2020).

Importantly, cavernomas then develop through clonal expansion of small populations of CCM‐deficient endothelial cells. Mosaic mouse models in which *CCM3/PDCD10* is deleted in a sparse, low‐frequency manner demonstrate that only a very small number of mutant endothelial cells are sufficient to seed a whole lesion, expand locally, and recruit surrounding wild‐type neighbors into the malformation (Detter et al. 2018).

In vitro co‐culture systems further support this concept: *CCM3* ^−/−^ endothelial cells outcompete wild‐type cells, induce mesenchymal‐like traits, and drive aberrant multicellular spheroid morphology. Interestingly, in cell culture experiments, endothelial cells deficient for CCM3 protein, but not wild‐type cells, show disproportionately strong growth suppression when exposed to p53‐restorative compounds such as NSC59984, highlighting a potential strategy to selectively target the mutant clone without harming the normal endothelium (Rath et al. 2022). Together, these observations support a model in which CCM originates from the clonal predominance of a few genetically “hit” endothelial cells, whose proliferative advantage and altered signaling landscape subsequently remodel the surrounding vascular niche into a mature CCM lesion.

All CCM lesions, irrespective of sporadic or familial origin, share a characteristic disorganization of interendothelial junctions within the neural vasculature. This structural instability allows chronic leakage and deposition of blood degradation products, typically visualized on MRI as a hypointense hemosiderin rim surrounding the lesion, an imaging hallmark of dysfunctional endothelial junctions and impaired barrier integrity (Tan et al. 2014; Tan et al. 2016). Histopathological analyses of surgically resected cavernomas confirm active endothelial angiogenesis and inflammatory cell infiltration, indicating that these lesions remain biologically active rather than quiescent (Sure et al. 2005; Girard, Zeineddine, Fam, et al. 2018). The best‐characterized cellular mechanisms underlying CCM pathophysiology are discussed in the subsections below.

### 3.2 Cell Adhesion Regulation by CCM Proteins

**FIGURE 2:**
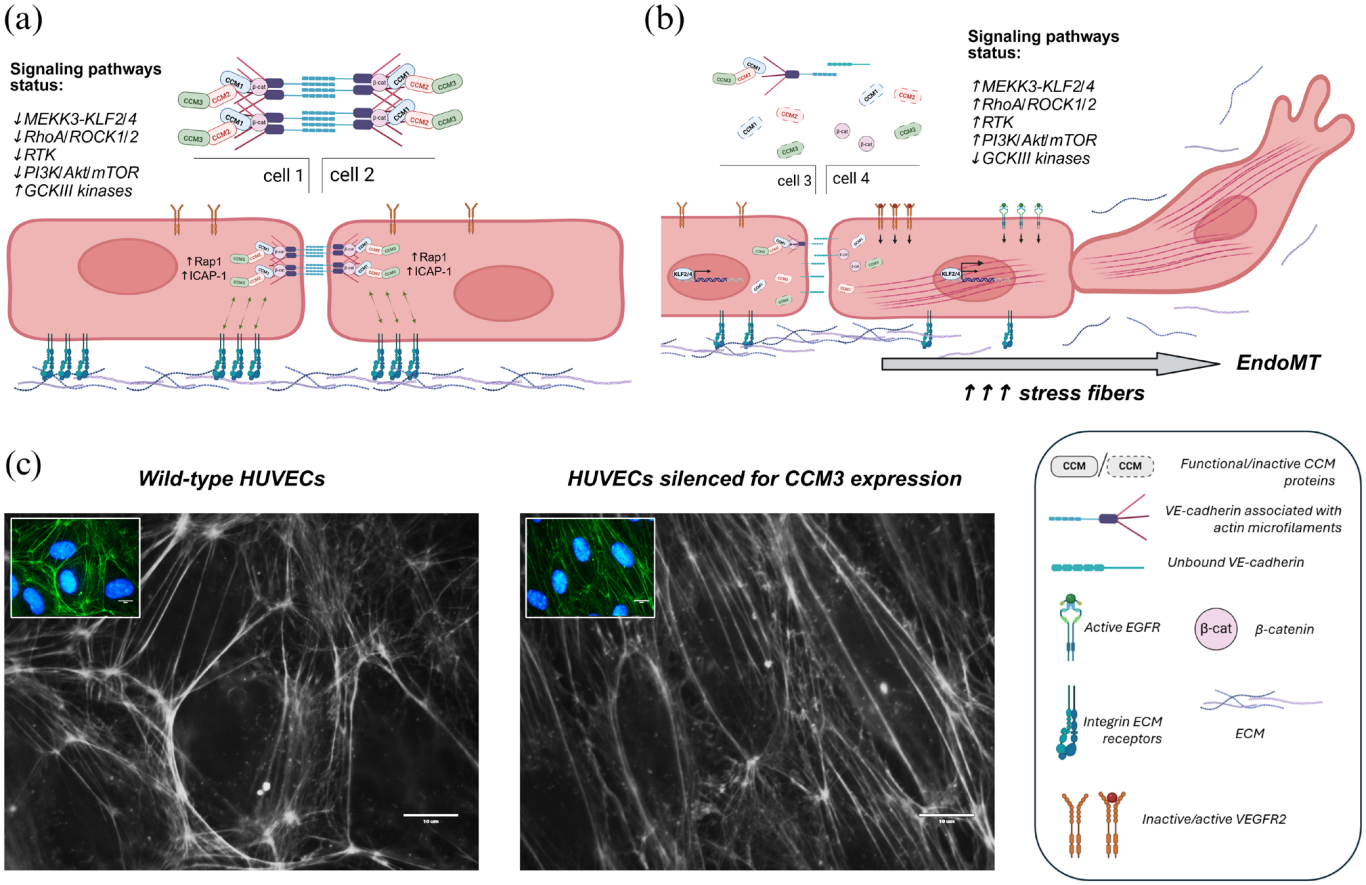
Key signaling pathways involved in cavernoma pathogenesis. (a) In quiescent brain endothelium, functional cerebral cavernous malformation (CCM) proteins interact with VE‐cadherin, catenins, and other junctional components to ensure the structural stability of the blood–brain barrier (BBB). A coordinated crosstalk between cell–cell and cell–matrix adhesion mechanisms (indicated by green arrows) involves Rap1, an adherens junction stabilizer, and ICAP‐1, an integrin inhibitor, acting in concert with CCM proteins. Together, these modulators maintain moderate RhoA/ROCK pathway activity, appropriate cortical tension, and low basal activity of the MEKK3–KLF2/4 signaling axis. CCM proteins—Particularly CCM3—Also restrain receptor tyrosine kinase (RTK) signaling, including VEGFR2 and EGFR, while preserving adequate activity of GCKIII kinases required for endothelial lumen homeostasis. (b) Loss‐of‐function mutations in any CCM gene result in dysfunctional CCM proteins and disassembly of adherens junctions, thereby activating the MEKK3–KLF2/4 pathway and increasing RhoA expression. Enhanced β1‐integrin signaling sustains persistent RhoA/ROCK activity, promotes stress fiber–dependent contractility, and drives extracellular matrix (ECM) remodeling. RTK hyperactivation has been described in CCM‐deficient endothelial cells, contributing to increased proliferation and impaired barrier integrity characteristic of CCM lesions. Endothelial‐to‐mesenchymal transition (EndoMT) has been associated with more aggressive familial and sporadic CCM variants. PI3K‐Akt–mTOR‐dependent signaling has been proposed as a somatic modifier that further fuels lesion progression. (c) Representative morphology of primary human endothelial cells illustrating the transition from a cortical Actin organization typical of junctional cells (left) to a stress fiber–rich phenotype in CCM3‐silenced cells (right). Actin microfilaments were stained with phalloidin–Alexa Fluor 488 (gray‐scale images; green highlights in the detailed boxes), and nuclei were counterstained with DAPI. Scale bar = 10 μm (*Source:* Unpublished data, LabAngio/UERJ).

CCM proteins act cooperatively and non‐redundantly to safeguard endothelial junctional stability, particularly through VE‐cadherin–associated adhesion complexes and the signaling networks that regulate them (Su and Calderwood 2020) (Figure 2a,b). Early work established hyperactivation of the RhoA/ROCK pathway as a pivotal determinant of the cavernous phenotype (Ayata et al. 2024). Although a direct biochemical interaction between CCM3 and RhoA/ROCK components has not been demonstrated, CCM3 forms a complex with VE‐cadherin, CCM1, and CCM2, and its loss induces the cortical‐to‐stress‐fiber cytoskeletal transition typical of cavernous endothelial cells in vitro (Voss et al. 2007) (Figure 2c).

Beyond these initial observations, CCM1 and CCM2 also regulate endothelial junctions by controlling the localization and activity of the small GTPase Rap1, a stabilizer of VE‐cadherin–mediated adhesion and an endogenous inhibitor of RhoA signaling (Lampugnani et al. 2017). Under physiological conditions, Rap1 promotes cohesive junctional architecture. However, when CCM1 or CCM2 function is impaired, Rap1 is displaced from the junctional complex, leading to unrestrained RhoA/ROCK activation, increased actomyosin contractility, and progressive loss of cell–cell adhesion. CCM proteins additionally influence other Rho‐family GTPases—such as Cdc42 and Rac1—key regulators of cytoskeletal organization and endothelial barrier function (Qi et al. 2023).

Endothelial homeostasis and barrier function result from the coordinated interaction among diverse adhesion receptors (Lampugnani et al. 2017). This interplay between intercellular adhesion and adhesion to the extracellular matrix (ECM) is crucial to endothelial instability in CCM. In vessel homeostasis, the integrin β1, a vital receptor for cell‐matrix adhesion, is also indispensable for the proper localization of VE‐cadherin to cell–cell junctions (Yamamoto et al. 2015). Rap1, a stabilizer of interendothelial junctions, also activates integrins in multiple cell types (Lampugnani et al. 2017). Loss of CCM1/2 disrupts barrier stability, leading to RhoA/ROCK hyperactivation due to enhanced upstream signaling from β1‐integrins (Faurobert et al. 2013; Renz et al. 2015). In CCM‐deficient cells, ICAP‐1, an inhibitor of β1‐integrin activation, becomes targeted for proteasome degradation, thereby exacerbating integrin signaling and augmenting RhoA‐dependent cytoskeletal tension (Faurobert et al. 2013). Notably, modulation of ECM composition can reverse cavernoma‐like phenotypes: both thrombospondin‐1 (TSP‐1) and fibronectin (FN) can restore junctional organization in CCM‐deficient endothelial cells (Lopez‐Ramirez et al. 2017; Schwefel, Spiegler, Kirchmaier, et al. 2020).

These findings underscore that CCM proteins coordinate adhesion across multiple structural layers—junctional, cytoskeletal, and ECM–integrin mediated—to maintain endothelial barrier function (Figure 2c).

### 3.3 The Kinase Axis of CCM‐Dependent Pathways

CCM appears to progress through intense dysregulation of cellular kinase activity, with recent evidence implicating additional signaling axes that are central to CCM pathophysiology. The MEKK3–MEK5–ERK5–KLF2/4 pathway is markedly upregulated in CCM‐deficient endothelial cells, where it functions upstream of RhoA/ROCK activation (Cuttano et al. 2016). Conditional loss of MEKK3 or KLF2/4 abolishes RhoA hyperactivation (Zhou et al. 2016). In homeostatic conditions, CCM2 directly binds MEKK3, likely restraining its activity; depletion of any CCM protein results in MEKK3 activation, nuclear accumulation of KLF2 and KLF4, and transcriptional induction of a maladaptive endothelial state (Fisher et al. 2015). KLF4 also drives endothelial‐to‐mesenchymal transition (EndoMT) in CCM models and clinical cavernomas (Maddaluno et al. 2013), a process enhanced by RhoA activity (Piera Velazquez and Jimenez 2019). This pathway provides a mechanistic explanation for both junctional fragility and abnormal morphogenesis.

Receptor‐tyrosine kinase (RTK) signaling further modulates CCM pathophysiology. Loss of CCM1, CCM2, or CCM3 destabilizes VEGFR2 regulation, leading to aberrant angiogenic signaling (DiStefano and Glading 2020). CCM3 stabilizes VEGFR2 through mechanisms involving receptor endocytosis (He et al. 2010), and CCM3‐deficient endothelial cells exhibit elevated EGFR levels (Sartages et al. 2020), suggesting a unique regulatory role for CCM3 in RTK homeostasis. These alterations may help explain the more severe clinical phenotype associated with *CCM3* mutations, especially in angiogenic or mitogenic microenvironments (Figure 2b).

Downstream of RTKs, the PI3K/AKT/mTOR pathway contributes to vascular remodeling and survival (Karar and Maity 2011). Although appropriate activation is necessary for endothelial viability and homeostasis, persistent AKT1 signaling induces abnormal vascular morphogenesis (Phung et al. 2006). Initially considered a defining feature of sporadic CCM, where somatic activating mutations in *PIK3CA* occur in the absence of germline *CCM* gene defects, *PIK3CA* gain‐of‐function (GOF) mutations have now also been identified across all three genotypes of familial CCM (Hong et al. 2021; Weng et al. 2021). These GOF mutations, which have also been considered powerful modifiers of CCM pathogenesis (see Section 3.4, below), drive excessive, abnormal vascular proliferation, suggesting that PI3K pathway activation contributes to the acquisition of clonal growth competence within CCM lesions, regardless of whether the initiating event is familial (germline) or sporadic.

CCM3 directly binds and stabilizes the GCKIII (Germinal Center Kinase III) kinase subfamily (MST3, MST4, and STK25), anchoring them within the STRIPAK complex and regulating endothelial polarity, junctional stability, and cytoskeletal dynamics. CCM3–GCKIII axis plays a central role in lumen formation (Qi et al. 2023). In vivo, loss of heterozygosity for either CCM3/*PDCD10* or *GCKIII* produces vessels with narrowed or irregular lumina, accompanied by dilation of adjacent microvascular segments, strikingly reminiscent of the cavernous, thin‐walled architecture characteristic of CCM lesions (Chan et al. 2011).

### 3.4 Immunoinflammatory and Genetic Modifiers of Cavernoma Progression

Emerging evidence highlights roles for inflammatory pathways and genetic modifiers (Tang et al. 2017; Starke et al. 2017). Variants affecting *TLR4* gene and mucosal barrier regulators have been proposed to modulate lesion burden, particularly in the context of CCM3‐related disease (Tang et al. 2017). Experimental work has directly implicated gut microbiome, innate immunity, and gut barrier integrity in CCM pathogenesis. In a seminal study, Tang et al. showed that endothelial Toll‐like receptor 4 (TLR4) stimulation by lipopolysaccharide derived from Gram‐negative gut bacteria is required to activate the MEKK3–KLF2/4 pathway and drive lesion formation in CCM mouse models: germ‐free mice or animals with genetic or pharmacologic blockade of TLR4 were protected from CCM development, whereas activation of TLR4 or polymorphisms increasing *TLR4* or *CD14* expression were associated with higher lesion burden in humans (Tang et al. 2017).

Building on this work, a subsequent study demonstrated that CCM3/PDCD10 has distinct roles in brain endothelium and gut epithelium, and that disruption of the colonic mucus barrier, via gut epithelial PDCD10 loss, Mucin‐2 deficiency, dietary emulsifiers or chemical injury, markedly increases CCM burden in *PDCD10*‐deficient mice (Tang et al. 2019). In that model, dexamethasone potently reduced CCM formation by acting on both brain endothelial and gut epithelial cells. Together, these data define a gut–brain disease axis in CCM, in which microbiota‐derived signals and gut barrier integrity modulate MEKK3–KLF2/4 activation and lesion formation, particularly in the context of *PDCD10* deficiency.

Interestingly, patients with active cavernomas (87% presenting with hemorrhage and 13% with seizure crises) showed increased circulating Th17/Tc17 subsets expressing functional TLR2 and, more prominently, TLR4, along with elevated memory B‐cell populations (Castro et al. 2022). These findings further underscore the contribution of the inflammatory axis to CCM pathogenesis.

Finally, although causal relationships remain unproven, vitamin D is increasingly viewed as a potential modifier of CCM progression. Observational studies demonstrate that low vitamin D levels correlate with aggressive or hemorrhagic disease, while supplementation has been associated with reduced future hemorrhage risk (Girard et al. 2016; Flemming et al. 2020; Previch et al. 2022). These clinical findings align with known endothelial effects of vitamin D, anti‐inflammatory signaling, reduced adhesion molecule expression, and enhanced tight junction stability (Equils et al. 2006; Song et al. 2023; Lazzara et al. 2022; Schröder Heurich et al. 2019), which conceptually fit the barrier‐disruptive mechanisms implicated in CCM lesions.

Together, these data support a model in which CCM proteins orchestrate a complex network involving junctional adhesion, integrin–ECM signaling, mechanotransduction, cytoskeletal regulation, kinase pathways (MEKK3–KLF2/4 and RhoA/ROCK), RTK signaling, GCKIII, inflammation, and transcriptional reprogramming. Disruption of this network destabilizes the endothelial barrier and creates a microenvironment prone to hemorrhage, inflammation, and dysfunction of the neurovascular unit.

## 4 CCM Mutations: Implications for Testing and Counseling

### 4.1 Overview of CCM Mutations

To date, 565 genetic variants have been identified in the *CCM* genes, of which 281 are classified as pathogenic and 25 as likely pathogenic, according to the current *ClinVar* public database (Landrum et al. 2025), by searching the entries “CCM, cavernoma” (https://www.ncbi.nlm.nih.gov/clinvar/?term=ccm+cavernoma). Globally, studies suggest that 40%–65% of pathogenic variants occur in *CCM1*, 15%–20% in *CCM2*, and 10%–15% in *CCM3* (Akers et al. 2025; Spiegler et al. 2018; Riant et al. 2013).

Pathogenic variants in *CCM1/KRIT1*, *CCM2/MGC4607*, and *CCM3/PDCD10* span a broad mutational spectrum, most of which converge on a loss‐of‐function mechanism. The majority are nonsense, frameshift, canonical splice‐site mutations, or large exon‐level deletions, which generate truncated transcripts subject to nonsense‐mediated decay and result in absent or severely reduced protein production (Riant et al. 2013; Bergametti et al. 2005). Missense variants, although less frequent, can disrupt essential structural domains, such as the KRIT1 NPxY motifs, the CCM2 PTB domain, or dimerization interfaces of PDCD10, thereby destabilizing the KRIT1–CCM2–PDCD10 complex and impairing protein–protein interactions required for normal endothelial signaling (Voss et al. 2007; Faurobert et al. 2013; Gault et al. 2005). Across all three genes, these molecular defects lead to dysregulated endothelial junctions and aberrant activation of MEKK3–KLF2/4 and RhoA/ROCK pathways, providing a unified mechanistic basis for lesion formation (Cuttano et al. 2016; Zhou et al. 2016). Thus, despite their genetic heterogeneity, most CCM mutations produce functionally equivalent cellular effects, consistent with haploinsufficiency as the dominant mechanism in familial CCM (Akers et al. 2025).

Regional genetic patterns in familial CCM are increasingly well characterized, with several well‐established founder mutations reported across populations. Four identified founder mutations have been reported, in addition to other recurrent mutations worldwide (Table 1) (Günel et al. 1996; Sahoo et al. 1999; Cavé Riant et al. 2002; Laurans et al. 2003; Cau et al. 2009; Gallione et al. 2011; Denier et al. 2006; Mondéjar et al. 2014; Ortiz et al. 2007; Liquori et al. 2007; Gallione et al. 2022; Cigoli et al. 2014; Guclu et al. 2005). A typical example is the Hispanic *KRIT1* founder mutation, which is highly prevalent in the American Southwest yet notably absent from the Iberian Peninsula, suggesting a New‐World–specific ancestral origin (Ortiz et al. 2007; Choquet et al. 2013). A distinct *KRIT1* founder variant has been identified in Sardinian families (Cau et al. 2009); a recurrent splice‐site *CCM2* mutation is also reported among Ashkenazi Jewish probands (Gallione et al. 2011). Meanwhile, in *CCM2*, a large exon 2–10 germline deletion has recently been confirmed as a North‐American founder mutation, shared by multiple apparently unrelated kindreds, a finding established through haplotype analysis and genealogical reconstruction, in a 2022 study (Gallione et al. 2022). In Brazil, sequencing of blood samples from 23 symptomatic and asymptomatic FCCM patients identified germline pathogenic variants in *CCM1* (53%), *CCM2* (33%), and *CCM3* (14%) (Galvão, da Silva, et al. 2024; Galvão, Trefilio, et al. 2024), underscoring the importance of locally driven efforts to characterize the genetic epidemiology of FCCM.

**TABLE 1:** Representative founder and recurrent familial cerebral cavernous malformation (CCM) mutations.

Gene	Variant	Type/molecular impact	Founder/recurrence context	Typical phenotype	Penetrance/severity	Key references
CCM1 (KRIT1)	c.1363C>T (p.Gln455Ter, Q455X)	Nonsense, truncating	*Common Hispanic founder* (Mexico, US Southwest)	Multiple lesions; seizures; hemorrhage; cutaneous venous lesions in subset	High but incomplete; variable expressivity	(Günel et al. 1996; Sahoo et al. 1999; Laurans et al. 2003)
CCM1	c.742C>T (“Mexican–American founder”)	Nonsense	*Founder mutation* in Mexican‐American lineages	Multiple CCMs; seizures; hemorrhage; headaches	Reduced penetrance; intrafamily variability	(Günel et al. 1996; Sahoo et al. 1999; Cavé Riant et al. 2002)
CCM1	C329X (p.Cys329Ter)	Nonsense	*Sardinian founder*	Typical familial CCM; multiple lesions; seizures/headache	High radiologic penetrance	(Cau et al. 2009)
CCM2 (MGC4607)	c.30+5_30+6delGCinsTT	Splice variant	*Ashkenazi Jewish founder*	Multiple CCMs; seizures; hemorrhage	High MRI penetrance	(Gallione et al. 2011)
CCM2	c.30+1G>A	Splice donor loss	Recurrent in Europe	Multiple lesions; seizures/hemorrhage	High but incomplete; classic “two‐hit” proven	(Pagenstecher et al. 2009; Denier et al. 2006; Mondéjar et al. 2014)
CCM2	14‐bp deletion in exon 5 (~aa230 trunc.)	Frameshift	*Iberian founder* (Spain/Portugal)	Multiple CCMs; seizures/hemorrhage	High radiologic penetrance; variable symptoms	(Mondéjar et al. 2014; Ortiz et al. 2007)
CCM2	77.6‐kb deletion (exons 2–10)	*Large germline deletion*, complete loss‐of‐function (LOF)	*Strong North‐American founder*, demonstrated by haplotype genealogy	Classic FCCM2: multiple CCMs; seizures; hemorrhage; variable severity	High penetrance; common in US clinical testing	(Liquori et al. 2007; Gallione et al. 2022)
CCM3 (PDCD10)	c.103C>T (p.Arg35Ter)	Nonsense	Recurrent truncation in multiple global cohorts	Early onset, high lesion burden; hemorrhage; spinal lesions	Very aggressive vs CCM1/2	(Shenkar et al. 2015; Bergametti et al. 2005; Cigoli et al. 2014)
CCM3	c.283C>T (p.Arg95Ter)	Nonsense	Recurrent (Europe, US, Asia)	Early hemorrhage; many lesions; *multiple meningiomas* in some	High penetrance; often symptomatic in childhood	(Shenkar et al. 2015; Riant et al. 2013; Cigoli et al. 2014; Guclu et al. 2005)
CCM3	Frameshifts cluster (exons 5–7)	Frameshift → LOF	Recurrent “hotspot class” globally	*Most severe CCM subtype*: early onset, high hemorrhage risk, spinal involvement, meningiomas	Very high penetrance	(Shenkar et al. 2015; Riant et al. 2013; Bergametti et al. 2005; Cigoli et al. 2014)

### 4.2 Genetic Testing and Counseling

The 2025 consensus guidelines recommend obtaining a three‐generation family history and performing genetic testing for *CCM1*, *CCM2*, and *CCM3* in patients with multiple cavernomas and no other predisposing factors (e.g., prior brain irradiation or developmental venous anomaly‐DVA) (Akers et al. 2025). MRI is the diagnostic modality of choice and ideally includes a susceptibility‐sensitive sequence (SWI, SWAN, or T2* GRE) to maximize lesion detection. Gadolinium enhancement is typically not required unless tumor or other vascular malformations are in the differential diagnosis.

**FIGURE 3:**
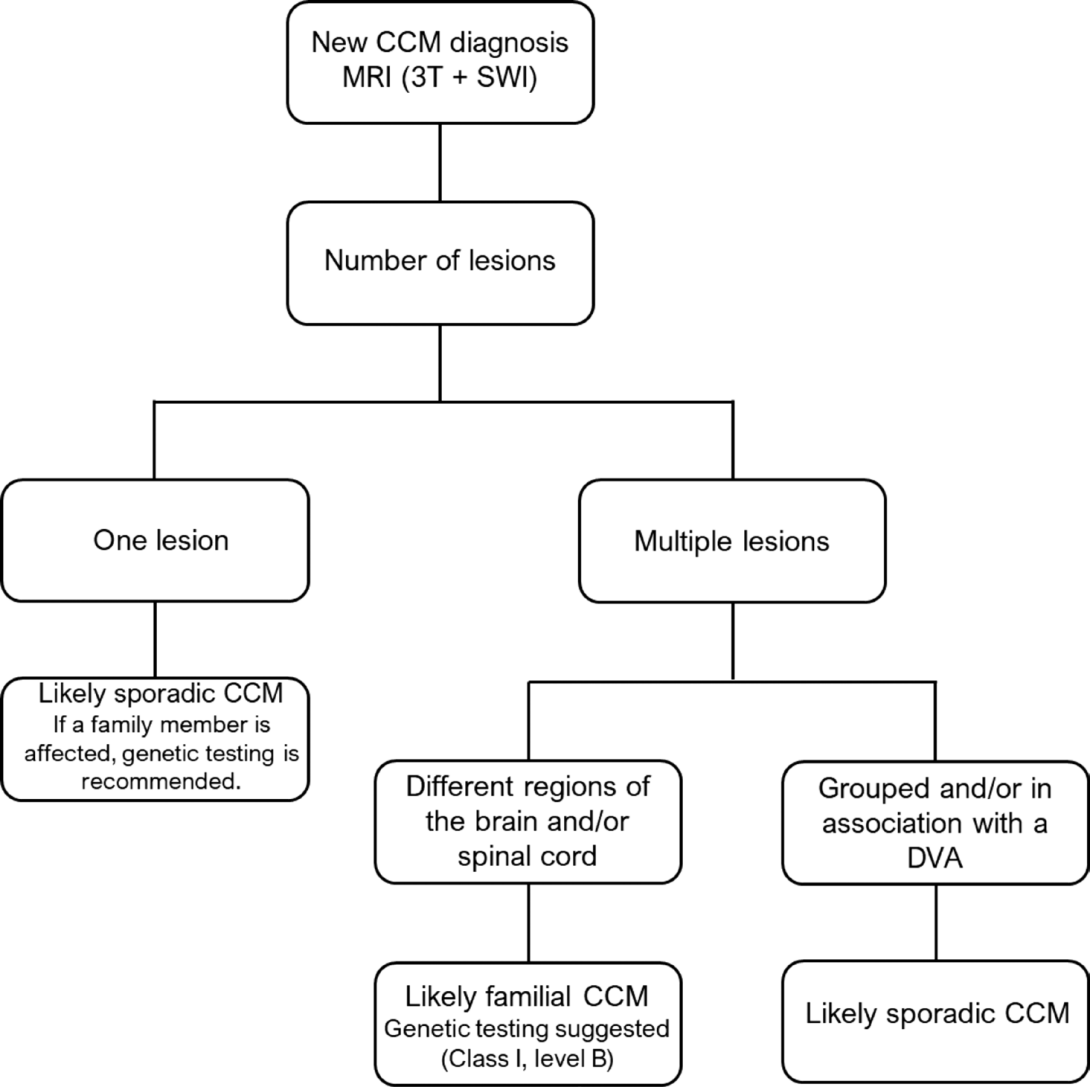
A flowchart of the investigation and management of a newly diagnosed cerebral cavernous malformation (CCM). Susceptibility‐weighted imaging (SWI) sequence is a hallmark for the differential diagnosis of sporadic CCM and familial CCM (FCCM). DVA, developmental venous anomaly; MRI (3 T + SWI), magnetic resonance imaging with susceptibility‐weighted imaging/3 Tesla magnet.

Figure 3 summarizes a current flowchart for guiding clinical investigation and counseling of potential mutation carriers and their susceptible families (de Souza et al. 2008; Akers et al. 2025). Consultation with a clinical geneticist is highly recommended. The diagnostic yield of molecular testing depends heavily on patient selection criteria, as discussed by Akers et al., and on testing methodology (Akers et al. 2025). Available techniques, Sanger sequencing, next‐generation sequencing (NGS), and MLPA, can identify point mutations, deletions, duplications, and other pathogenic variants, though none provides perfect sensitivity (Spiegler et al. 2018; Riant et al. 2013). FCCM3 is clinically distinct, with a more aggressive course, earlier hemorrhage, and associated features such as scoliosis, benign CNS tumors, and cognitive disability, reported in approximately 60% of cases (Shenkar et al. 2015; Galvão, da Silva, et al. 2024).

Approximately half of individuals with FCCM remain asymptomatic throughout life, although this estimate may be an underestimate of the true prevalence because many carriers are never imaged (Flemming et al. 2023; Denier et al. 2006). Evidence suggests that recurrence risk correlates with the degree of genetic relatedness, as shown in studies involving twins, siblings, and extended biological relatives (Santos et al. 2022; Dammann et al. 2013; Chang et al. 2019; Schröder et al. 2014; Haasdijk et al. 2012). The absence of symptoms cannot rule out FCCM in at‐risk relatives (Haasdijk et al. 2012). Predictive testing can clarify risk for family members, potentially excluding a familial pathogenic variant. Still, it should be accompanied by counseling because genetic information may increase anxiety or depression in some individuals (Schlich Bakker et al. 2006; Cicero et al. 2017). Genetic screening is not recommended when a patient presents with a single lesion and no affected relatives (Schröder et al. 2014).

Importantly, patients must understand that a negative genetic test does not exclude FCCM. Because autosomal dominant inheritance may still occur, first‐degree relatives remain at risk. Approximately 10%–20% of families with a clear autosomal dominant pattern of CCMs remain negative for pathogenic variants in *CCM1/KRIT1*, *CCM2*, and *CCM3/PDCD10*, even after comprehensive sequencing and copy‐number variant (CNV) analysis (Flemming et al. 2023; Akers et al. 2025; Scimone et al. 2017).

These so‐called gene‐negative familial CCM families are clinically indistinguishable from *CCM1*‐ or *CCM2*‐related disease but lack the aggressive, early‐childhood, brainstem‐predominant phenotype typical of *CCM3/PDCD10* carriers (Shenkar et al. 2015; Riant et al. 2013). This subset of clinically sporadic patients may present with multiple CCM lesions and their gene‐negative status most likely reflects limitations of standard genetic testing, including: deep intronic or regulatory variants not captured by routine exome or panel testing; complex structural variants or genomic rearrangements missed by standard CNV pipelines; high‐level mosaicism or low‐level somatic mutations confined to CCM lesions, which may not be detected in blood‐derived DNA, detectable only with ultra‐deep sequencing (Shenkar et al. 2015; Akers et al. 2009; Riant et al. 2013; Scimone et al. 2017; McDonald et al. 2014; Sikta et al. 2025). In such cases, molecular testing can be highly informative by identifying the causative defect, enabling more accurate diagnostic classification and guiding genetic counseling for the patient and offspring. Accordingly, current international recommendations support offering genetic testing to any individual with multiple CCM lesions, even in the absence of known affected family members (Akers et al. 2025).

Less commonly, individuals with multiple bilateral lesions but no identifiable mutation may harbor pathogenic variants in non‐coding regions or in an as‐yet‐unidentified fourth *CCM* gene, which current methods cannot detect (Labauge et al. 2007; Spiegler et al. 2018; Riant et al. 2013). The last newly identified *CCM* gene was *CCM3*, 20 years ago (Bergametti et al. 2005). The recent identification of copy‐number‐neutral genomic rearrangements involving *CCM2*, using whole‐genome sequencing, has made the “fourth‐gene hypothesis” rather unlikely (Spiegler et al. 2018).

### 4.3 Pregnancy and Pediatric Counseling

For females with FCCM considering pregnancy, early consultation, preferably with a genetics specialist, is crucial. Counseling should address reproductive options including preimplantation genetic testing and prenatal screening, allowing informed decisions about family planning (Santos et al. 2022; Joseph et al. 2021). Although reports remain limited, both the literature and our institutional experience indicate that requests for prenatal diagnosis are common, especially among individuals with family members who experienced severe disease manifestations. Patients should be informed that mutation carriers may remain asymptomatic and that current knowledge does not alter available therapeutic interventions (Bacigaluppi et al. 2013). Technological advances have made prenatal and preimplantation genetic diagnosis feasible for known familial mutations, thereby improving the detection of embryonic or fetal abnormalities and refining embryo selection for in vitro fertilization (IVF) (Vermeesch et al. 2016). Prior studies highlight the importance of these discussions: in familial pediatric cases, penetrance of *CCM* mutations has been reported at 85%, with 68% experiencing hemorrhagic events (Merello et al. 2016).

During pregnancy, all typical CCM symptoms require close monitoring. A pregnant patient with suspected FCCM should receive genetic counseling to understand fetal risks and available options. Severe headaches or new focal neurological deficits warrant a prompt MRI to avoid missing a potential cerebral hemorrhage. Pregnancy‐related epilepsy also requires careful evaluation. A frequent concern involves the safest mode of delivery. Although theoretical risk exists, evidence suggests that pregnancy and vaginal delivery do not increase hemorrhage rates (Akers et al. 2025; Joseph et al. 2021; Cauldwell et al. 2022). Continuation of antiepileptic medications is generally advised, as fetal risks from these drugs are usually outweighed by the dangers of uncontrolled maternal seizures. Drug levels may require adjustment throughout pregnancy.

Approximately 25% of FCCM cases occur in the pediatric population (Geraldo et al. 2023). A recent multicenter study of 41 children from four tertiary pediatric centers reported a 5‐year annual symptomatic bleeding rate of 5%, comparable to rates seen in adults with sporadic or familial CCM (Geraldo et al. 2023). Zafar and colleagues recommend genetic testing in children whenever treatment decisions are being considered (Zafar et al. 2019).

## 5 Biomarkers

Because CCM is clinically heterogeneous and lesion activity is often unpredictable, reliable biomarkers are essential for improving patient surveillance and risk stratification. Objective molecular or imaging markers could identify subclinical lesion instability, clarify prognosis, and enable the selection of more homogeneous, high‐risk cohorts for clinical trials. This would substantially strengthen trial design and accelerate therapeutic development.

### 5.1 Imaging Biomarkers

MRI was decisive in characterizing the typical ‘popcorn‐like’ lesion, which results from multiple signals emitted by blood in varying states of degradation and is surrounded by hemosiderin deposits. To enhance CCM detection sensitivity, Gradient Echo (T2*/GRE) sequences were developed and revealed the “blooming” effect of hemosiderin, an artifact expanding the CCM image that accentuates hemosiderin deposits (de Souza et al. 2008; de Champfleur et al. 2011).

Souza et al. demonstrated in 2008 that SWI sequences were more sensitive than T2*/GRE images for detecting multifocal lesions in familial forms of the disease (Figure 1b,c). This sequence revealed more than twice as many lesions as conventional MRI, which is considered the gold standard for FCCM diagnosis and is a diagnostic imaging biomarker (de Souza et al. 2008; de Champfleur et al. 2011). Because the leakage of hemoglobin from the lesion is a hallmark of the disease, and because iron deposits are present in the nervous tissue surrounding a CCM, a novel magnetic resonance application uses quantitative susceptibility mapping (QSM) to assess correlations with clinical features in humans (Tan et al. 2014; Girard et al. 2017). QSM was reported to improve accuracy in iron content in the CCM, and a 6% increase in QSM threshold was shown to reflect recurrent symptomatic hemorrhage (Castro et al. 2022; Girard et al. 2017).

### 5.2 Fluid Biomarkers

Using hierarchical clustering analysis, higher plasma levels of IL‐1β and sROBO4, combined with lower levels of sCD14, IL‐6, and VEGF, were observed in individuals who experienced a second hemorrhage at one‐year follow‐up. A weighted combination of these four molecules predicted symptomatic hemorrhage with 86% sensitivity and 88% specificity, representing one of the earliest prospective attempts to stratify risk in CCM patients (Girard et al. 2016; Girard, Zeineddine, Koskimäki, et al. 2018). More recently, extensive multiplex proteomic studies in familial CCM have strengthened this concept, identifying additional circulating biomarkers associated with disease presence, genotype, severity, and progression (Li et al. 2025). Reduced CD31 and BDNF levels distinguished FCCM patients from unaffected first‐degree relatives, whereas low PAI‐1 and elevated ROBO4 correlated with greater lesion burden and recurrent bleeding.

Other inflammatory and endothelial markers, including sCD14, LBP, CXCL4, ICAM‐1, ANG2, THBS1, CCL5, CRP, and decreased HDL, were significantly altered in symptomatic FCCM, with subsets such as sENG, THBS1, and CXCL4 showing genotype‐specific patterns (Lazzaroni et al. 2023). Likewise, sROBO4, thrombomodulin, and CRP predicted incident clinical events (ICH, focal neurological deficits, or seizures). At the same time, GDF‐15, FLT3L, CXCL9, FGF‐21, and CDCP1 were associated with the emergence of new MRI‐detectable lesions over two‐year follow‐up.

Three complementary studies demonstrated that plasma biomarkers reflecting inflammation and endothelial disruption can stratify hemorrhagic risk in CCM. In one study, a weighted four‐analyte panel (sCD14, IL‐1β, VEGF, sROBO4) predicted symptomatic hemorrhagic expansion (hemorrhage or lesional growth) within 1 year with 86% sensitivity, 88% specificity, and an AUC of 0.90, validated in an independent cohort (Girard, Zeineddine, Koskimäki, et al. 2018). A parallel investigation focusing specifically on CASH (cavernous angioma with symptomatic hemorrhage) identified partially overlapping signatures: a diagnostic panel (sCD14–VEGF–CRP–IL‐10) distinguishing recent CASH with 76% sensitivity and 80% specificity, and a prognostic panel—again converging on sCD14, VEGF, IL‐1β, and sROBO4—predicting future hemorrhage with 83% sensitivity and 93% specificity (Lyne et al. 2019). Another recent report linked changes in circulating molecules such as ROBO4, CD14, thrombomodulin, endoglin, TSP2, and IL16 (TM) to imaging changes reflecting new symptomatic hemorrhages during the prospective follow‐up of CCM patients (Hage et al. 2025). These studies, using distinct outcome definitions and biomarker‐selection strategies, collectively establish a reproducible multi‐marker axis associated with lesion instability in CCM.

Complementary proteomic analyses of human CCM tissue further revealed signatures of endothelial activation, leukocyte infiltration, ECM remodeling, and astrocyte–microglial activation, with biomarkers such as CD93, ICAM‐1, and MMP9 linked to hemorrhagic lesions and validated in murine models (Jauhiainen et al. 2024). Together, these studies support the growing view that circulating and tissue‐derived biomarker signatures reflect the inflammatory and angiogenic dysregulation underlying lesion growth and rupture.

In parallel, the same research groups have demonstrated that miRNA expression profiles mirror the underlying germline mutation in both mouse models and human patients (Romanos et al. 2023), suggesting that easily accessible circulating miRNA assays may help identify individuals at risk for developing new lesions, providing a promising non‐invasive tool for prognostic applications in FCCM (Alcazar Felix, Jhaveri, et al. 2025).

## 6 Disease Management

Once careful clinical, radiological, and genetic assessment has identified affected patients and their relatives, disease management becomes the central focus, requiring tailored surveillance, symptom‐directed therapies, and multidisciplinary decisions regarding surgical or emerging medical interventions.

### 6.1 Neurological and Neurosurgical Management

There are currently no formal guidelines for follow‐up imaging in FCCM, particularly for individuals who remain asymptomatic. Surveillance must therefore be individualized, with repeat MRI obtained when new neurological symptoms or signs raise concern for recent lesion activity. Patients with CCM3 mutations, given their more aggressive biological behavior, warrant closer clinical and radiological monitoring. Conversely, in FCCM cases identified during familial screening, when lesions are small, asymptomatic, and incidentally discovered. A conservative approach with reassurance about the typically benign course is appropriate (Awad and Polster 2019; Maraire and Awad 1995; Gault et al. 2006).

In individuals with epilepsy related to CCM, surgical intervention can offer reasonable postoperative seizure control (von der Brelie et al. 2014). However, most FCCM patients with seizures can be managed conservatively: approximately 50%–60% achieve seizure freedom with antiepileptic therapy alone. Imaging the seizure focus is unnecessary unless the patient presents with difficult‐to‐control epilepsy (DCE) and is being evaluated for potential lesional resection (Akers et al. 2025). As with other chronic neurological conditions, management should balance the natural history of the disease with risks associated with intervention. Surveillance includes monitoring symptoms, optimizing seizure control, and implementing rehabilitative measures when stable neurological deficits persist (Batra et al. 2009).

The decision to proceed with surgical resection remains complex despite updated guideline recommendations. Surgical risk must always be weighed against the lesion's expected natural history (Akers et al. 2025; Batra et al. 2009). In multifocal FCCM, the decision should focus on the symptomatic lesion, with surgery typically reserved for (i) first or recurrent hemorrhage in accessible regions, (ii) progressive neurological deterioration, (iii) medically intractable epilepsy when the epileptogenic focus is clear, or (iv) mass effect with symptoms. Surgery is not recommended for asymptomatic lesions, particularly those in deep or brainstem locations, except in cases of refractory epilepsy (Awad and Polster 2019; Moultrie et al. 2014).

Deep or eloquent‐area CCMs carry substantial operative risk: postoperative morbidity ranges from 5% to 18%, with mortality around 2%, even in highly experienced centers (Gross et al. 2009). Spinal CCMs are particularly challenging, and surgical decisions must carefully weigh surgeon expertise and the natural history of the disease, as intervention remains controversial (Akers et al. 2025; Jehi et al. 2014; Rosenow et al. 2013). Additionally, because FCCM patients often harbor multiple lesions, surgical cure is inherently limited, and recurrence or *de novo* lesion formation may complicate long‐term outcomes.

Given these considerations, observational management, regular clinical assessment, MRI surveillance when appropriate, and treatment of symptoms, is frequently favored for asymptomatic or minimally symptomatic familial lesions. While avoiding the risks of surgery, observation demands vigilance to detect lesion progression or hemorrhage. The presence of a genetic predisposition adds complexity: new lesions may arise over time, requiring periodic reassessment of the risk–benefit balance for continued conservative management (Santos et al. 2022).

### 6.2 FCCM and Radiation Therapy

Cranial radiation therapy (CRT) is associated with the late development of radiation‐induced cavernous malformations (RICMs), particularly in pediatric cancer survivors. Most RICMs remain asymptomatic, and lesion formation typically appears years after exposure, with one report indicating a median onset of 8 years post‐CRT (Cutsforth Gregory et al. 2015). In a retrospective cohort of 239 patients treated with CRT three decades earlier, 10 CCM cases were identified radiologically, with a median age of 8.7 years at the time of CRT; all had received doses > 30 Gy, suggesting a dose‐dependent risk of CCM emergence (Gastelum et al. 2015).

There is also evidence that CRT may exacerbate underlying FCCM biological risks. A report documented a loss‐of‐function CCM1 variant in a surgical specimen from a child with a dense cluster of RICMs 7 years after CRT; the child's asymptomatic father carried the same mutation but had only two small lesions, implying that radiation may act as a second hit, worsening the familial disease phenotype (Russo et al. 2017). Systematic sequencing of CCM lesions arising after CRT may help clarify the interaction between radiation‐induced vascular injury and FCCM genetic susceptibility.

A recent study found a striking enrichment of *CCM1* mutations, with four involving exon 5, suggesting a potential radiation‐sensitive hotspot (Tsutsumi et al. 2025). Only 1 of 46 non‐irradiated patients carried a *CCM1* mutation, reinforcing the contrast. The latency to lesion detection averaged 31 years, and several patients also developed radiation‐associated meningiomas. Despite the limited sample size, these findings support a mechanism distinct from hereditary FCCM and point to exon 5 of *CCM1* as unusually susceptible to radiation‐induced damage.

### 6.3 Additional Surgical and Observational Considerations

Although surgery has long been practiced in CCM, its effectiveness in FCCM, particularly in hemorrhagic cases, remains debated, as no high‐quality trials provide definitive guidance. The appropriateness of resection should be considered based on lesion size, anatomical location, prior clinical events, and overall neurological impact (Awad and Polster 2019). A population‐based study highlighted poor long‐term outcomes following surgery for deeply located lesions in the insula, basal ganglia, thalamus, or brainstem, reinforcing the importance of careful patient selection (Gross et al. 2009). Observational management remains a cornerstone for asymptomatic lesions. However, clinicians must remain aware that FCCM predisposes patients to ongoing lesion genesis, which may modify risk over time. Clinical decisions should therefore be revisited periodically, incorporating new imaging findings and symptom evolution.

Finally, radiosurgery, though effective for solitary sporadic cavernous malformations, is not recommended in FCCM due to the risk of radiation‐induced *de novo* lesions, as reflected in the Guidelines for the Clinical Management of Cerebral Cavernous Malformations (Class III, Level C evidence) (Akers et al. 2025). Given these risks, radiosurgery should be considered only in very select circumstances where the anticipated benefit clearly outweighs the potential harm.

### 6.4 Quality of Life

The functional scales traditionally used to assess patients with CCM and other neurological conditions often fail to capture aspects of daily functioning and overall well‐being that matter most to patients. For this reason, patient‐reported outcomes (PROs), which reflect the individual's perception of symptoms, functional limitations, and treatment impact, have gained increasing importance in both clinical practice and research. PROs provide a complementary perspective to conventional neurological assessments, offering insight into the multidimensional experience of living with CCM (Lapin et al. 2019; Arwert et al. 2022; Kumar et al. 2019).

Few studies have evaluated health‐related quality of life (HRQoL) in CCM using validated instruments. Our research group was among the first to address this knowledge gap, demonstrating that patients managed conservatively, particularly those who were initially considered for surgery but ultimately not treated, can maintain a good quality of life (Bicalho et al. 2017). These early findings underscored the potential disconnect between structural lesion burden and subjective well‐being.

Subsequent studies have expanded this understanding. Herten et al. investigated 219 untreated CCM patients. They found that HRQoL was reduced compared with the general population, with the strongest impairments observed in anxiety and depression, even in patients without neurological deficits (Herten et al. 2021). Similarly, researchers at the Amsterdam University Medical Center examined HRQoL in 205 untreated CCM patients and found a significant reduction relative to Dutch population norms, particularly in mental health domains. They also identified clinical correlates of poorer HRQoL, including a history of symptomatic hemorrhage, epilepsy, and brainstem lesions, all of which were associated with more substantial functional and emotional impact (Sandmann et al. 2025).

Building on these findings, Kim et al. evaluated patients who had experienced symptomatic hemorrhage from CCM within the previous year. They found that at least 30% reported worsened HRQoL, particularly marked by elevated anxiety relative to reference populations (Kim et al. 2021). Within the Treat_CCM cohort, HRQoL analysis revealed a significant reduction in the physical component score. Although anxiety levels were not elevated, clinically significant depression was present in 31.7% of participants, emphasizing the burden of chronic symptoms and uncertainty experienced by these patients (Meessen et al. 2024).

More recently, our group conducted the first study directly FCCM and sporadic (SCCM) forms using the PROMIS‐29 and EQ‐5D‐5L instruments. In a cohort of 83 patients, we found no significant differences in HRQoL between FCCM and SCCM, challenging the assumption that the genetic form inherently results in worse patient‐perceived outcomes (Cunha et al. 2025). Instead, HRQoL disparities were driven primarily by symptom status rather than genetic subtype: among fCCM patients, those who were symptomatic reported significantly worse scores for usual activities (EQ‐5D‐5L; *p* = 0.014) and physical function (PROMIS‐29; *p* = 0.031) than asymptomatic individuals. These findings reinforce the concept that clinical presentation, not underlying genotype, is the dominant determinant of perceived HRQoL in CCM.

Altogether, the growing body of HRQoL research in CCM demonstrates that psychological burden, functional limitation, and fear of future hemorrhage play an essential role in patient well‐being. The integration of PROs into routine care and clinical trials will be essential for capturing meaningful treatment effects and improving patient‐centered outcomes.

## 7 Perspectives in Experimental Models and Drug Development

### 7.1 Preclinical Therapeutic Landscape in FCCM

#### 7.1.1 Cell‐Based Models

A broad spectrum of in vitro systems has been developed to model CCM pathophysiology, ranging from simple endothelial monolayers to complex organoid platforms. Traditional 2D cultures using primary or immortalized endothelial cells (HUVECs, HBMECs, murine ECs) remain foundational for dissecting *CCM* gene‐loss‐of‐function effects, including junctional instability, RhoA/ROCK hyperactivation, KLF2/4 upregulation, and mechanotransduction defects (as shown in Figure 2) (Renz et al. 2015; Cuttano et al. 2016; Zhou et al. 2016; Whitehead et al. 2009). These tools have been refined by generating isogenic CRISPR‐edited endothelial lines with targeted knockouts of *CCM1*, *CCM2*, or *CCM3*, enabling precise genotype–phenotype analyses (Lopez‐Ramirez et al. 2017; Li, Tran, et al. 2021; Schwefel, Spiegler, Much, et al. 2020).

Co‐culture systems in which wild‐type and *CCM*‐deficient endothelial cells are grown together have revealed non‐cell‐autonomous effects, including clonal expansion of mutant cells, altered contact‐dependent signaling, and competitive dynamics that mimic early lesion mosaicism (Detter et al. 2018; Rath et al. 2022; Zhou et al. 2016; Malinverno et al. 2019).

Although 3D culture systems better capture the architectural complexity of cavernomas, microfluidic microvessel‐on‐chip models—up‐and‐coming for studying CCM dynamics, are still rare in the field. Induced pluripotent stem cell (iPSC) platforms have recently emerged as robust human‐based systems to model CCM pathogenesis. An iPSC line derived from a healthy donor (UNCCi002‐A) served as a versatile wild‐type control for CCM research, enabling standardized comparisons between mutant and non‐mutant endothelial lineages (Beltran et al. 2021). Complementing these findings, a CRISPR‐engineered *CCM1* knockout iPSC model revealed that loss of KRIT1 during endothelial differentiation elicits a distinct and reproducible gene‐expression signature, including dysregulation of pathways involved in cell–cell adhesion, angiogenic signaling, and cytoskeletal organization (Pilz et al. 2023). This in vitro system confirms that KRIT1 loss directly drives endothelial phenotypes central to cavernoma biology.

Recent advances in human stem‐cell–based systems have generated powerful preclinical models of CCM. Using high‐throughput differentiation of blood‐vessel organoids from *CCM1*, *CCM2*, or *CCM3* knockout hiPSCs, both shared and mutation‐specific defects were demonstrated across endothelial, neuronal, mesenchymal, and fibroblast populations (Skowronek et al. 2025). Mosaic blood vessel organoids revealed abnormal clonal expansion of *CCM1*‐ and *CCM3*‐deficient cells, whereas *CCM2*‐deficient cells showed reduced proliferation, immediately suggesting a cellular basis for the milder clinical course typically associated with *CCM2* variants.

Through notable methodological advances, two groups developed iPSC‐derived human blood–brain barrier (hBBB) assembloids (or organoids) that combine brain and vascular organoids derived from a human embryonic stem cell lineage (Dao et al. 2024). These assembloids faithfully recapitulated cavernoma architecture and BBB disruption, and transcriptomic comparisons between assembloids and human lesion tissue revealed convergent CCM‐related molecular alterations. This model provides unprecedented resolution of neurovascular crosstalk in CCM and directly links patient‐specific endothelial defects to BBB failure.

In the second study, the first to develop a patient‐derived iPSC model of CCM, it was demonstrated that heterozygous *CCM1* mutations alone are sufficient to trigger endothelial abnormalities, even before the acquisition of a second hit (Arce et al. 2025). Upon endothelial differentiation, these *CCM1* ^+/−^ iPSCs generated vascularized organoids with cavernoma‐like dysmorphic structures, exhibited disrupted junctional organization, and showed transcriptional alterations—including increased expression of *PEG3*, a progenitor/stress‐response gene also elevated in human familial and sporadic CCM lesions. When introduced into ex vivo brain explants, the mutant endothelial cells integrated abnormally and induced focal vascular anomalies, reinforcing the relevance of early endothelial dysfunction in CCM.

Together, these three iPSC‐based platforms represent an expanding toolkit for dissecting CCM mechanisms in human cells, capturing early pathogenic events, identifying endothelial transcriptional programs, and enabling controlled modeling of gene‐specific perturbations.

#### 7.1.2 Animal Models of CCM

Several complementary animal models have been essential for elucidating CCM biology and for advancing therapeutic development. Neonatal endothelial‐specific knockout mice, created by tamoxifen‐inducible deletion of *CCM1*, *CCM2*, or *CCM3* in early postnatal endothelium, develop highly penetrant cerebellar and retinal cavernomas within weeks, faithfully reproducing hemorrhage, barrier defects, and KLF2/4 and RhoA/ROCK pathway activation (Zhou et al. 2016; Whitehead et al. 2009). Although powerful for mechanistic discovery, early lethality limits long‐term studies.

Another range of animal models has been developed to study CCM lesion initiation, progression, and molecular mechanisms. Endothelial‐specific, inducible mouse knockouts remain the gold standard for modeling human disease. Deletion of *CCM1*, *CCM2*, or *CCM3* in neonatal endothelial cells, typically using *PDGFB‐CREERT2*, *CDH5‐CREERT2*, *SLCO1C1‐CREERT2* drivers, produces brain lesions that closely resemble human cavernomas, with robust activation of MEKK3–KLF2/4 signaling, RhoA/ROCK hyperactivation, and increased vascular permeability (Zhou et al. 2016; Detter et al. 2020; Cardoso et al. 2020; Lopez Ramirez et al. 2019). Among these, *CCM3* models exhibit the most aggressive phenotype, characterized by early onset, high lesion burden, and elevated hemorrhage rates, consistent with the clinical severity of CCM3. Adult‐onset conditional models (e.g., brain‐endothelial *CCM3* deletion, *PDCD10* ^*ECKO*^ mice) generate chronic, multifocal lesions with prolonged survival, allowing longitudinal MRI and biomarker evaluation (Zeineddine et al. 2019; Alcazar Felix, Shenkar, et al. 2025). Another interesting model (*Map3k3* ^*I441M*^ */Cdh5‐creER* ^*T2*^) crossbred mice introduced the first endothelial‐specific *Map3k3* ^*I441M*^ knock‐in mouse model that closely mirrors the genetic configuration observed in sporadic human CCMs, in which a single heterozygous somatic mutation in *MAP3K3* is frequently detected. Unlike previous overexpression‐based models, this approach preserves physiological gene dosage and endothelial specificity, substantially improving clinical relevance (Xu et al. 2025). These models have been instrumental in evaluating pharmacological strategies to limit hemorrhage and to target PI3K/mTOR signaling in both familial and sporadic CCM.

Additional insights derive from two‐hit “mutator” models, in which a germline heterozygous CCM mutation is combined with impaired DNA repair (e.g., *CCM1* ^+/−^, *MSH2* ^−/−^, *CCM2* ^+/−^; *TRP53* ^−/−^). These mice stochastically acquire somatic second hits, generating multifocal lesions that established the genetic basis of clonal endothelial expansion and provided first proof‐of‐concept that fasudil reduces lesion burden (McDonald et al. 2012; Gibson et al. 2015).

Finally, zebrafish models with *CCM1*, *CCM2*, or *CCM3* mutations enable high‐resolution imaging of vascular defects, flow‐dependent mechanotransduction, and rapid drug screening. Zebrafish studies have validated key CCM pathways and supported pharmacologic targets such as β‐adrenergic blockade and ROBO4/THBS1 signaling (Lazzaroni et al. 2023; Li, Shenkar, et al. 2021).

Together, these animal models, from rapid zebrafish screens to chronic adult murine systems, provide a coherent experimental framework for dissecting CCM pathogenesis and testing mechanistically informed therapies.

### 7.2 Drug Repositioning and Development

The investigation of FCCM and SCCM increasingly incorporates considerations of therapeutic repositioning. Some observational studies suggest an association between lower vitamin D levels and increased CCM lesion activity; however, no evidence supports the use of vitamin D supplementation as a preventive strategy (Akers et al. 2025).

Symptomatic patients are generally advised to avoid nonsteroidal anti‐inflammatory drugs (NSAIDs), whereas asymptomatic individuals may use them cautiously. Antithrombotic and anticoagulant medications are traditionally avoided except in life‐threatening situations under close monitoring. Recent observational data indicate a potential protective effect of antithrombotic therapy against hemorrhage in CCM (Zuurbier et al. 2019), but this has not yet been incorporated into formal guidelines (Akers et al. 2025). Moreover, a cohort study suggested an increased risk of hemorrhage in CCM patients using female hormonal therapies, including contraceptives and menopausal hormone treatments; caution is advised until more robust risk stratification is possible (Zuurbier et al. 2023).

Among repositioned drugs, β‐blockers have recently entered the clinical arena. The Italian TREAT_CCM trial, the first randomized interventional study explicitly conducted in familial CCM, evaluated the safety and potential activity of propranolol. This randomized, open‐label, blinded‐endpoint phase 2 pilot trial demonstrated that propranolol was safe and well tolerated and reported a possible signal of efficacy, with fewer clinical events (symptomatic hemorrhage or focal neurological deficit) in the propranolol group than in the standard care group (Lanfranconi et al. 2023). These findings prompted interest in planning a future phase 3 trial.

A contemporaneous commentary raised methodological considerations relevant to interpreting the Treat_CCM pilot findings (Shenkar and Awad 2023). As expected in early‐phase rare‐disease trials, the small number of clinical events limits the precision of estimated treatment effects, and aspects of outcome classification and the open‐label design may have introduced variability in symptom reporting. Practical issues such as heterogeneous dosing, variable drug exposure, and differences in vitamin D supplementation further illustrate the challenges inherent to conducting interventional studies in FCCM. A complementary preclinical report also noted that the human‐equivalent doses used in Treat_CCM were lower than those associated with biological effects in murine models, underscoring the broader need for careful dose‐finding in translational research (Shenkar et al. 2022). These considerations do not diminish the significance of Treat_CCM as a pioneering pilot study; instead, they underscore the complexity of therapeutic development in FCCM and the importance of future double‐blind, biomarker‐informed studies with optimized dosing strategies to assess propranolol's potential more definitively.

Futhermore, the AT CASH EPOC randomized, double‐blind, placebo‐controlled trial evaluated high‐dose atorvastatin (80 mg/day) for 2 years in adults who had experienced symptomatic hemorrhage within the prior year (Awad et al. 2025). Despite a strong preclinical rationale, atorvastatin did not alter lesional iron deposition on quantitative susceptibility mapping (QSM) MRI and did not reduce symptomatic rebleeding compared with placebo. The drug was safe and well‐tolerated, and the trial established a valuable framework for QSM‐based biomarker endpoints in CCM therapeutic trials. Taken together, the propranolol and atorvastatin studies illustrate both the promise and the challenges of translating preclinical discoveries into effective CCM pharmacotherapies.

A second drug‐repositioning strategy evaluated simvastatin, motivated by its pleiotropic endothelial‐stabilizing properties and its ability to modulate vascular inflammatory signaling. In the completed phase I study “Permeability MRI in Cerebral Cavernous Malformations Type 1 Patients: Effects of Statins” (NCT01764451), dynamic contrast–enhanced MRI–based permeability measurements were employed as surrogate endpoints to assess biological activity. In the corresponding pilot randomized controlled trial, simvastatin did not produce a measurable reduction in lesion permeability compared with placebo. Nevertheless, the study provided valuable proof of concept and methodological experience with quantitative permeability imaging biomarkers for CCM clinical trials (Mabray et al. 2020).

A substantial body of preclinical evidence implicates the RhoA/ROCK pathway as a central mediator of endothelial dysfunction in CCM, making ROCK inhibition a particularly promising therapeutic avenue. ROCK activation has been demonstrated across multiple CCM genotypes and correlates with lesion development and maturation (Montagnoli et al. 2023). In murine models of aggressive CCM3 disease, treatment with the clinically approved ROCK inhibitor fasudil, as well as atorvastatin, significantly reduced lesion burden, hemorrhage, inflammation, and endothelial proliferation compared with placebo, whereas simvastatin did not show similar benefit (McDonald et al. 2012; Shenkar et al. 2019). Fasudil also reduced lesion burden in *CCM1/MSH2* and *CCM2/TRP53* mice, accompanied by decreased expression of ROCK‐activation biomarkers.

Targeting ROCK2 specifically yielded parallel effects: mice with ROCK2 hemizygous deletion showed reduced lesion burden, and the selective ROCK2 inhibitor BA‐1049 (now known as NRL‐1049) decreased lesion number and size in *CCM1*‐ and *CCM3*‐mutant mice (McKerracher et al. 2020). A phase 1 study examining the safety, tolerability, and pharmacokinetics of NRL‐1049 in healthy volunteers found that the maximum tolerated dose of 150 mg was associated with a favorable safety profile (Madden et al. 2025).

In parallel with these in vivo findings, recent bioinformatics work by our group has expanded the landscape of potential ROCK‐targeting compounds (Franco et al. 2025). Using a structure‐guided pharmacophoric map of ROCK1/2, virtual screening identified several FDA‐approved agents, most notably the JAK inhibitors ruxolitinib and baricitinib, as previously unrecognized, highly potent ROCK inhibitors (IC_50_ values in the low‐nanomolar range), with tivozanib exhibiting marked ROCK2 selectivity. Molecular modeling revealed conserved hinge‐binding interactions, and ruxolitinib demonstrated anti‐inflammatory effects in glial assays, supporting the biological relevance of ROCK inhibition in neurovascular contexts. These findings provide additional chemical scaffolds and repurpose candidates that may complement or refine future CCM‐directed ROCK‐modulation strategies.

Beyond β‐blockers and statins, additional drug‐repositioning initiatives and first‐in‐disease programs have progressed into early‐phase clinical evaluation for CCM. One mechanistically distinct drug‐repositioning strategy targets pathological remodeling of the extracellular matrix. Doxycycline, a tetracycline antibiotic with well‐characterized matrix metalloproteinase (MMP)–inhibitory properties, has been evaluated in a phase 1 clinical context in brain vascular malformations (Influence of Matrix Metalloproteinase on Brain Arteriovenous Malformation Hemorrhage; NCT00783523). The underlying rationale is supported by clinicopathological evidence demonstrating robust endothelial expression of MMP‐2 and MMP‐9 in surgically resected CCM lesions (Fujimura et al. 2007) and by pilot translational studies showing that short‐term doxycycline administration suppresses MMP‐9 levels in resected arteriovenous malformation tissue (Hashimoto et al. 2005). Although arteriovenous malformations and CCM represent distinct neurovascular entities, convergent evidence of MMP upregulation in hemorrhage‐prone lesions provides a biologically plausible translational rationale for evaluating MMP‐modulatory strategies in CCM.

More recently, REC‐994 (also referred to as tempol), an orally bioavailable redox‐cycling nitroxide designed to attenuate superoxide‐driven oxidative stress, advanced through randomized phase 1 dose‐escalation and dose‐finding studies in healthy volunteers. These studies demonstrated approximately dose‐proportional systemic exposure across a broad dosing range and a favorable tolerability profile compatible with chronic administration (Alfa et al. 2024). This development enabled initiation of the phase 2 SYCAMORE trial in patients with symptomatic CCM (NCT05085561). Initial public disclosures indicated that the study met prespecified safety and tolerability endpoints and suggested exploratory signals at the 400‐mg dose after 12 months of treatment. However, subsequent analyses from the long‐term extension phase reportedly failed to sustain these preliminary trends, with no meaningful improvements in MRI‐based or functional outcomes beyond those anticipated in the absence of active treatment, ultimately leading to discontinuation of the SYCAMORE program. Despite its termination, the trial contributed substantially to establishing the multicenter CCM clinical trial infrastructure. It underscored the necessity of durable, biomarker‐aligned efficacy signals when advancing therapeutic candidates in rare neurovascular disorders.

In parallel, immune‐targeting approaches have begun to enter clinical investigation. Daratumumab, a first‐in‐class anti‐CD38 monoclonal antibody approved for the treatment of multiple myeloma, induces depletion of CD38‐expressing plasma cells and can remodel immune‐cell compartments through multiple effector mechanisms (Sanchez et al. 2016). Its evaluation in familial CCM (NCT07026604) is supported by evidence that CCM lesions may harbor antigen‐driven, oligoclonal intralesional IgG responses, together with broader signatures of immune activation (Shi et al. 2009; Zhang et al. 2020). Conceptually, this trial marks a shift away from purely hemodynamic or endothelial‐barrier–centric interventions toward testing whether modulation of pathogenic immune components can alter lesion activity or clinical event rates in genetically defined CCM populations.

**FIGURE 4:**
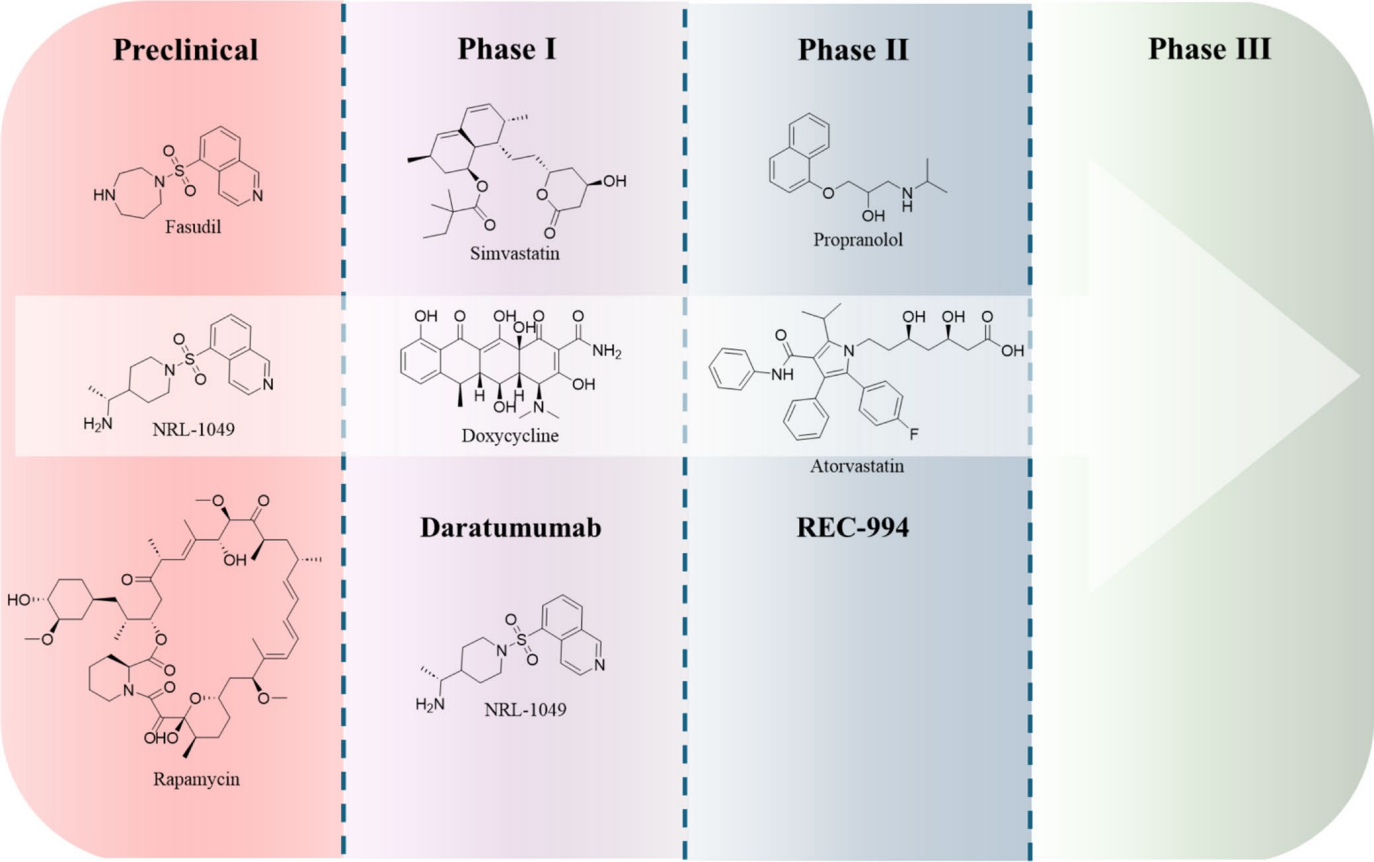
Therapeutic pipeline for cerebral cavernous malformation (CCM): Clinical and preclinical candidates. Although significant advances have been made in defining the molecular basis of cavernoma disease, translation into late‐phase (Phase II/III) clinical trials remains limited. The schematic highlights the drugs that have been tested or are under investigation for CCM treatment. Statins (simvastatin, atorvastatin) exert pleiotropic endothelial‐stabilizing and anti‐inflammatory effects. Fasudil and NRL‐1049 inhibit RhoA/ROCK signaling, thereby reducing cytoskeletal tension and vascular dysfunction. Rapamycin targets PI3K–Akt–mTOR signaling, which is implicated in lesion progression. REC‐994 is an orally active small molecule that acts as an antioxidant scavenger and has been shown to reduce lesion burden in preclinical models. Propranolol, a non‐selective β‐adrenergic antagonist, has been explored for its vascular‐modulating properties.

An overview of ongoing and recently completed clinical repositioning efforts, along with emerging mechanistically driven candidates, is presented in the CCM therapeutic pipeline (Figure 4). Future progress will rely on integrating genotype‐stratified trial designs with quantitative imaging and molecular biomarkers to enable mechanism‐informed patient selection and robust evaluation of therapeutic efficacy.

## 8 Concluding Remarks and Future Perspectives

The evidence reviewed here highlights several emerging hypotheses in FCCM pathogenesis and clear avenues for future investigation. Current models increasingly view lesion formation as the result of interplay between germline loss‐of‐function mutations and endothelial stress responses, including inflammatory, oxidative, and mechanotransducive pathways, which may help explain the substantial variability in penetrance and lesion burden across individuals. Parallel advances in neuroimaging and molecular profiling suggest that integrating quantitative MRI metrics with circulating or tissue biomarkers could ultimately transform FCCM into a condition defined by objective radiological and molecular signatures.

Given the growing evidence of RhoA/ROCK pathway hyperactivation, particularly in CCM3‐associated disease, the possibility that pharmacologic modulation of this axis may offer therapeutic benefit warrants rigorous preclinical and early‐phase clinical evaluation. Progress in the coming decade will depend on coordinated international efforts to assemble large, deeply phenotyped cohorts, validate candidate biomarkers, and harmonize clinical outcome measures.

Finally, because FCCM affects patients and families in ways that extend beyond neurological morbidity, systematic investigation of psychosocial and quality‐of‐life dimensions remains an essential component of a comprehensive, patient‐centered research agenda.

## Author Contributions

**Fabrícia Lima Fontes‐Dantas:** conceptualization, writing – original draft. **Gustavo da Fontoura Galvão:** writing – original draft. **Alexandre Martins Cunha:** writing – original draft. **Pedro de Sena Murteira Pinheiro:** writing – original draft. **Verônica Morandi:** conceptualization, writing – original draft, supervision, writing – review and editing. **Jorge Marcondes de Souza:** conceptualization, writing – original draft, writing – review and editing, supervision.

## Funding

The authors have been funded by grants and fellowships from the following Brazilian organisms: (i) CNPq/National Council for Scientific and Technology Development—Ministry of Science, Technology and Innovation; (ii) Foundation for the Support of Scientific Research of the State of Rio de Janeiro (FAPERJ), Rio de Janeiro State Government; (iii) CAPES/Coordination for the Improvement of Higher Education Personnel, Ministry of Education.

## Consent

The authors have nothing to report.

## Conflicts of Interest

The authors declare no conflicts of interest.

## Data Availability

Data sharing does not apply to this article because no new data were created or analyzed for this review.
